# Serum Vitamin D Level and Risk of Community-Acquired Pneumonia: A Case-Control Study

**DOI:** 10.1155/2021/2157337

**Published:** 2021-11-28

**Authors:** Guwani Liyanage, Anusha Kaneshapillai, Suthesan Kanthasamy

**Affiliations:** ^1^Department of Paediatrics, Faculty of Medical Sciences, University of Sri Jayewardenepura, Nugegoda, Sri Lanka; ^2^Department of Biochemistry, Faculty of Medical Sciences, University of Sri Jayewardenepura, Nugegoda, Sri Lanka; ^3^Paediatric Professorial Unit, Colombo South Teaching Hospital, Kalubowila, Sri Lanka

## Abstract

**Introduction:**

Recent research has shown conflicting evidence on the connection between vitamin D deficiency and community-acquired pneumonia (CAP) in children. Thus, we hypothesized that vitamin D deficiency could be a risk factor for CAP.

**Methods:**

Hospitalized children between 2 and 60 months with physician-diagnosed, radiologically confirmed severe community-acquired pneumonia (CAP) were enrolled as cases. Age-matched controls were enrolled from immunization and weighing clinics. A blood sample was collected to assess serum 25-(OH)D concentration. Unconditional logistic regression was done to examine the independent association of vitamin D level with community-acquired pneumonia.

**Results:**

Seventy-four children (females: 68%) were included. Overall, 27% had vitamin D deficiency (<20 ng/mL) and 37.8% had insufficiency (20–29 ng/mL). The vitamin D level ranged from 8.67 to 46.2 ng/mL. There was no statistically significant difference in 25(OH)D levels in controls and cases (*p*=0.694). In unconditional logistic regression, 25(OH)D concentration was not a determinant of CAP (OR: 0.99, CI: 0.937–1.044, *p*=0.689). This lack of association remained after adjustment for age, gender, income, crowding, and exposure to passive smoke (OR: 0.99, CI: 0.937–1.065, *p*=0.973). Household income was significantly associated with CAP (OR: 0.11, 95% CI: 0.021–0.567, *p*=0.008).

**Conclusion:**

Two-thirds of the children with CAP had vitamin D deficiency/insufficiency. In comparison with healthy controls, vitamin D level was not a significant determinant of community-acquired pneumonia. It informs that further multisite research is required using more rigorous scientific methods for conclusive evidence on the relationship between vitamin D and CAP.

## 1. Introduction

Vitamin D is a micronutrient important for bone growth. In addition, vitamin D receptors are ubiquitous and found in many other cell types and not confined to cells responsible for the bone and mineral metabolism [1]. Thus, it is suggested that vitamin D has immune-modulatory and antiinfective properties in addition to skeletal functions [1]. Vitamin D receptors can stimulate the expression of antibacterial peptides that resist bacterial and viral respiratory infections [2]. Also, recent studies have confirmed that vitamin D could directly regulate the innate and adaptive immunity, which is closely related with respiratory infections [3]. Many researchers have reported conflicting evidence on the relationship between serum vitamin D/vitamin D supplementation and CAP [4–7]. Berry et al. reported that respiratory tract infections incidence dropped by 7% when the serum vitamin D level increased by 4 ng/dL [6]. Another retrospective study demonstrated the likelihood of hospitalization due to CAP rose when the serum vitamin D level was <14.8 ng/dL [7]. Both these studies imply that vitamin D deficiency (VDD) may impact on CAP. On the other hand, Manaseki-Holland et al. reported that vitamin D supplementation is not an effective intervention to reduce the incidence of pneumonia in children [5].

South Asian countries, including Sri Lanka, have abundant sunlight throughout the year. However, prior research has shown a high prevalence of vitamin D deficiency among all age groups in this region [8–10]. With this background, we hypothesized that vitamin D deficiency could be a risk factor for CAP. Hence, this study compared vitamin D levels of children between 2 and 60 months with severe CAP and age-matched healthy controls.

## 2. Methods

### 2.1. Study Design and Sample Calculation

A matched case-control study was conducted in one large metropolitan hospital located in Colombo, Sri Lanka, between October 2018 and April 2019. This hospital is public-funded and serves a population of lower- and middle-income categories. The sample size was calculated using the following formula: *n* = 2(*Z*_*α*/2_ + *Z*_*β*_)^2^ (*σ*^2^/*d*^2^) [11]. When calculated for 80% power, *Z*_*β*_ is 0.84, and for 0.05 significance level, *Z*_*α*/2_ is 1.96. According to a previous study [12], the standard deviation was taken as 6.88 ng/dL (*σ* = 6.88), and the difference of vitamin D level expected was taken as 70% of the standard deviation (*d* = 4.8 ng/dL). Thus, the minimum sample size calculated was 32 for each group.

### 2.2. Procedure

Consecutive children (2–60 months) with physician-diagnosed pneumonia admitted for inward treatment were screened. Only those who met the inclusion criteria for severe CAP were invited for the study. Children with immune deficiency, hospital-acquired pneumonia, chronic disorders, history of pneumonia during the previous year, underlying severe cardiac or respiratory disorders, and children on vitamin D supplements during the previous three months were excluded. Age was considered as the matching factor related to CAP in children for study purposes. Thus, age-matched (within ± 12 months) healthy controls were recruited. This study was conducted following the Declaration of Helsinki and approval from the Ethics Committee of Sri Lanka College of Pediatricians (SLCP: 44/18). Written informed consent was taken from the parent/guardian of both cases and controls. Sample recruitment was carried out by an investigator who was not involved in patient management.

Information was gathered using an interviewer-administered, prepiloted questionnaire. Basic information on demography, total family income, previous health, breastfeeding, sun exposure time, vitamin supplements, and exposure to passive smoking was recorded. In addition, a physical examination was performed. Results of biological parameters were extracted from the hospital records (complete blood count and C-reactive protein). Children were followed up till discharge. Only the chest X-ray (CXR) confirmed CAP was included in the study. CXRs were reported by a consultant radiologist, who was blinded to the clinical data. In children with multiple CXRs, the final report was given after reviewing all. Age-matched controls were enrolled from immunization and weighing clinics in the same week as the case through convenient sampling.

A blood sample (3 ml) was collected from cases within the first 48 hours of admission and at enrollment from controls for vitamin D analysis. Serum was stored at −80°C until analysis. Total 25-(OH)D was measured using LIAISON® 25 OH Vitamin D TOTAL assay with chemiluminescent immunoassay technology.

### 2.3. Definitions

World Health Organization case definition was used to define the severity of community-acquired pneumonia [13]. Pneumonia was defined as cough or difficult breathing with tachypnoea and lower chest indrawing. Severe pneumonia was defined as the presence of any of the following features: oxygen saturation <90%, central cyanosis, grunting or very severe chest indrawing, inability to breastfeed or drink or vomiting everything, convulsions, lethargy, or reduced level of consciousness, decreased breath sounds or bronchial breath sounds, or signs of pleural effusion or empyema. Tachypnoea was defined as respiratory rate >50 breaths per minute for children aged 2–11 months and >40 breaths per minute for children aged 12–60 months. The World Health Organization criteria for interpreting radiographs were used for reporting uninterpretable, normal, end-point consolidation with or without pleural effusions or interstitial infiltrates [14]. The vitamin D status was assessed by measuring total 25-(OH)D. Concentration <20 ng/mL was categorized as vitamin D deficiency, 20–29 ng/mL as vitamin D insufficiency, and ≥30 ng/mL as sufficiency [15]. Crowding was defined if more than two individuals were sleeping in the child's bedroom. Exposure to cigarette smoke was defined if the parent/caregiver had noticed tobacco odor in the house during the previous two months. Household income was dichotomized (<43,000 vs.  ≥ 43,00) based on the median income per household in Sri Lanka [16]. The length of hospital stay measured the severity of CAP.

### 2.4. Data Analysis

Statistical analysis was performed using the SPSS Statistic version 22 software package. Data were checked for normality. Continuous variables were expressed as mean, standard deviation, median, and interquartile range (IQR), and categorical variables as frequencies and percentages. Mean differences and 95% confidence intervals (95% CI) were calculated with the independent sample *t*-test. Comparisons were performed with the chi-squared analysis and Mann–Whitney *U* test. Unconditional logistic regression was done to examine the independent association of vitamin D with community-acquired pneumonia [17]. Age was the design variable used in determining the sampling probabilities, and it was used at the premodelling stage and subsequently at the multivariate modeling stage. Other potential confounding covariates considered were gender (male vs. female), crowding (yes vs. no), exposure to passive smoke (yes vs. no), and household income (<43,000 vs.  ≥ 43,000 LKR). Crowding and household income were taken as proxy socioeconomic status variables. Outliers were cleared before multivariate regression.

## 3. Results

Seventy-four children were included in this case-control study (cases: 37 and controls: 37). Response rates were 100% for cases and 84% for controls. Overall, the mean age (SD) was 31.51 (18.5) months. Most were females (68%). Nearly one-quarter of the participants were from low-income families. The mean (SD) serum vitamin D level was 26.7 (8.5) ng/mL, and the range was between 8.67 and 46.2 ng/mL. Overall, 27% had vitamin D deficiency (<20 ng/mL) and 37.8% had insufficiency (20–29 ng/mL). Among cases, 27% and 40.5%, and among controls, 27% and 35.1% were deficient and insufficient. Mean vitamin D did not differ between cases and controls (mean difference: 0.79, CI: −3.24–4.84, *p*=0.694). Sun exposure time was not different between the two groups (*X*^*2*^: 3.091, *df* = 1, *p*=0.079).

Disease-related clinical and biological characteristics were analyzed (Table 1). The most common CXR finding was interstitial infiltrates. One child had a pleural effusion which was confirmed by ultrasonography. Two children required high dependency care. Most stayed five days in the hospital. The longest hospital stay was 30 days. No deaths were reported. Vitamin D category (deficient/insufficient vs. sufficient) concentration was not associated with the length of hospital stay (U: 95, *p*=0.214).

In unadjusted logistic regression, 25 (OH)D concentration was not a determinant of CAP (OR = 0.989, CI = 0.937–1.044, *p*=0.689) (Table 2). Children in the control group were from higher-income families than cases. Crowding was observed more among cases. In both groups, exposure to passive smoke was similar.

The lack of association of serum vitamin D with CAP remained after adjustment for age, gender, income, crowding, and exposure to passive smoking (0.99 (0.937–1.065), *p*=0.973) (Table 3). However, household income remained significant when adjusted for other variables. Odds of having CAP were approximately 11% greater for children with low-household income.

## 4. Discussion

We examined the relationship between serum vitamin D status and CAP among hospitalized children between 2 and 60 months with physician-diagnosed, radiologically confirmed severe community-acquired pneumonia. Although two-thirds of them had vitamin D deficiency/insufficiency, vitamin D level was not observed as a determinant of CAP in this population. Previous observational studies had reported conflicting results about the link between vitamin D and CAP. Most had observed an association than not [4, 7, 18]. In Nigeria, Oduwole et al. detected that vitamin D insufficiency (16–28 ng/mL) and not deficiency (<16 ng/mL) had a link to CAP in children [4]. Guo et al., in an age-matched case-control study, demonstrated that susceptibility and severity of viral pneumonia are significantly higher in children with vitamin D deficiency and insufficiency [18]. Yet, a randomized controlled trial in Afghanistan among 1–11 months old children revealed lack of any benefit of vitamin D supplementation on the incidence of pneumonia [5]. Due to the conflicting evidence, the World Health Organization has not recommended supplementation of children with vitamin D to prevent respiratory tract infections until further clarifications are done [19].

We did not find a connection between vitamin D status and the length of hospital stay, which was considered the measure of severity. However, previous studies have reported varied results regarding the connection between the severity of CAP and vitamin D status. Banajeh et al., using oxygen saturation as the marker of severity, demonstrated that vitamin D deficiency predicted oxygen saturation on day five [20]. A Cochrane review (2019) of seven randomized controlled trials on vitamin D supplementation and outcomes concluded that it is doubtful whether vitamin D has an important effect on CAP outcomes [21]. This review studied children from one month to five years hospitalized with acute community-acquired pneumonia. One explanation for varying results could be the lack of homogeneity of severity study designs.

Prior research has shown socioeconomic factors, access to care, crowding, air pollution, malnutrition, and lack of immunization as risk factors for pneumonia [22, 23]. A prospective population-based study on pneumonia in Brazil cited that those children from deprived socioeconomic conditions had a higher incidence of pneumonia [24]. In our cohort, family income was a determinant of CAP, which is a socioeconomic proxy variable. Furthermore, crowding, an indicator of socioeconomic status, is significantly associated with CAP cases when unadjusted. We considered the number of individuals sleeping in the child's bedroom as the indicator for crowding. Dwelling in crowded conditions encourages the spread of airborne pathogens [25]. Crowding had been defined differently in various studies: total number of individuals in the household, number of siblings, and number of individuals sharing the bed [25, 26]. We could have refined our findings more if multiple indicators were used.

Exposure to tobacco smoke was not a risk factor in our cohort. However, prior research has reported environmental tobacco smoke as a risk factor for pneumonia in children [27, 28]. We did not include the number of cigarettes smoked per day by the household member/s. The amount of passive smoking could be very different in each household, and it showed a direct dose-response relationship between the dose of smoking and lower respiratory infections in children [29].

Vitamin D deficiency/insufficiency was reported in two-thirds of children in our study. Degree of air pollution, sun exposure, type of residence, supplementation, and diet could affect vitamin D status [30, 31]. The study's design did not allow the analysis of all these factors except sun exposure. We found a lack of association between 25(OH)D and sun exposure time. This could probably be explained by not controlling for the time of the day and the area of the skin exposed to the sun. In Sri Lanka, vitamin D supplementation is not a part of the national nutrition protocol yet. Supplementation is not practiced in most tropical countries, like Sri Lanka, probably due to the common assumption that the level of solar radiation would ensure adequate vitamin D status [32].

Following are the strengths of our study. All the cases of pneumonia diagnosed in hospital settings by experienced pediatricians during the study period were screened for eligibility. We recruited only severe, radiologically confirmed pneumonia adhering to the standard World Health Organization criteria. However, there are few limitations in our study. First, case-control studies do not provide the best evidence. Eventhough they are not as powerful as other types of studies verifying causal relationships, case-control studies indicate the link between a risk factor and an outcome. Thus, they can direct future research. Second, this study was carried out in one center in an urban setting. Therefore, results cannot be generalized to all settings. Third, although the regression model was adjusted for comorbidities, other unmeasured confounders (e.g., use of biomass fuel and malnutrition) could affect the results.

## 5. Conclusions

Two-thirds of the children with CAP had vitamin D deficiency/insufficiency. In comparison with healthy controls, vitamin D level was not a significant determinant of community-acquired pneumonia. It informs that further multisite research is required using more rigorous scientific methods for conclusive evidence on the relationship between vitamin D and CAP.

## Figures and Tables

**Table 1:** Laboratory and radiological parameters of CAP.

Parameter	*n* (%)
CXR findings	
Consolidation ± effusion	15 (40.5)
Interstitial infiltrates	22 (59.5)
C-reactive protein (mg/L)	
>40	17 (45.9)
<40	20 (54.1)
WBC (10^9^/L), mean (SD)	17 (7.59)
Neutrophils (10^9^/L), mean (SD)	11 (6.91)
^*∗*^Hospital-stay (days), median (Q1, Q3)	07 (5, 10)

Values are presented as number *n* (%) unless otherwise indicated. CAP, community-acquired pneumonia; CXR, chest X-ray; SD, standard deviation; WBC, white cell count.

**Table 2:** Bivariate analysis of factors associated with community-acquired pneumonia.

	Cases, *n* (%)	Controls, *n* (%)	OR	95% CI	*P* value
Age^*∗*^ (months)	29.68 (17.4)	33.35 (19.6)	0.989	0.965–1.014	0.391
Sex					
Male	20 (54.1)	14 (37.8)	1.0		
Female	17 (45.9)	23 (61.1)	0.541	0.213–1.373	0.196
Household income (LKR)					
<43,000	15 (40.7)	2 (5.4)	1.0		
≥43,000	21 (56.3)	35 (94.6)	0.080	0.017–0.385	0.002
Crowding					
No	11 (29.7)	01 (02.7)	1.0		
Yes	26 (70.3)	36 (97.3)	0.066	0.008–0.541	0.011
Smoking					
No	28 (75.7)	30 (81.1)	1.0		
Yes	09 (24.3)	07 (18.9)	1.378	0.452–4.196	0.573
Serum 25(OH)D^*∗*^ (ng/mL)	27.08 (9.57)	26.28 (7.57)	0.989	0.937–1.044	0.689

^*∗*^Mean (SD). CI, confidence interval; LKR, Sri Lankan rupees; OR, odds ratio.

**Table 3:** Multivariate analysis of factors associated CAP with unconditional logistic regression taking cases and controls as the dependent variable.

	Coefficient *β*	Exp (B)	95% CI for exp (B)		*P* value
			Lower	Upper	
^*∗*^Serum 25(OH)D, ng/mL	−0.001	0.999	0.937	1.065	0.973
^*∗*^Age (months)	−0.002	0.998	0.969	1.029	0.920
Female	−0.306	0.736	0.243	2.233	0.589
Household income >43,000 LKR	−2.212	0.109	0.021	0.567	0.008
Crowding	−2.185	0.112	0.011	1.106	0.061
Smoking	0.290	1.337	0.361	4.956	0.664

^*∗*^Continuous variables. Log-likelihood ratio = 76.82, Nagelkerke *R*^2^ = 0.35, *X*^2^ = 21.58, *p*=0.001. CI, confidence interval; LKR, Sri Lankan rupees.

## Data Availability

The data used to support the findings of this study are available from the corresponding author upon request.

## Abbreviations

BMI: Body mass index
CAP: Community-acquired pneumonia
C.I: Confidence interval
CXR: Chest X-ray
LKR: Sri Lankan rupees
IQR: Interquartile range
WHO: World Health Organization.
